# The Performance of Two Rapid Antigen Tests During Population-Level Screening for SARS-CoV-2 Infection

**DOI:** 10.3389/fmed.2021.797109

**Published:** 2021-12-23

**Authors:** Mohammad Alghounaim, Hamad Bastaki, Farah Bin Essa, Hoda Motlagh, Salman Al-Sabah

**Affiliations:** ^1^Department of Pediatrics, Amiri Hospital, Ministry of Health, Kuwait City, Kuwait; ^2^COVID-19 Research Team, Jaber Alahmad Hospital, Ministry of Health, Kuwait City, Kuwait; ^3^Department of Public Health, Ministry of Health, Kuwait City, Kuwait; ^4^Department of Surgery, Faculty of Medicine, Kuwait University, Kuwait City, Kuwait

**Keywords:** COVID-19, SARS-CoV-2, antigen test, population surveillance, immunoassay

## Abstract

**Background:** SARS-CoV-2 antigen assays offer a rapid mean to diagnose and isolate infected individuals. However, their utility in population-level screening is unknown.

**Objectives:** The performance of two antigen tests in detecting SARS-CoV-2 was assessed among individuals randomly selected in the community.

**Study Design:** A prospective study that performed head-to-head comparison of two SARS-CoV-2 antigen assays. Individuals were recruited during community SARS-CoV-2 screening over 10 working days. Demographic and clinical data were collected. Standard Q COVID-19 Ag test, a point-of-care chromatographic assay, was conducted immediately, and then the sample was transported to the virology laboratory to perform PCR and the LIAISON SARS-CoV-2 Ag chemiluminesence immunoassay.

**Results:** respiratory samples from 991 individuals were collected, and 62 were positive by PCR. Inconclusive PCR results were observed in 19 samples and were excluded. The median age of participants was 40.2 years (IQR 32.3–47.8), and 932 (94%) were males. Most (77.4%) of infections were asymptomatic. The sensitivity and the specificity of the LIAISON assay were 43.3% (95%CI 30.6–56.8) and 99.9% (95%CI 99.3–100). The Standard Q assay had lower sensitivity (30.6%, 95%CI 19.6–43.7) but similar specificity (98.8%, 95%CI, 97.8–99.4). Similarly, the LIAISON assay had higher positive predictive value (96.3%, 95%CI 81–99.9% vs. 63.3%, 95%CI, 43.9–80.1%). Both assays performed better in symptomatic patients and among samples with a low-cycle threshold (Ct < 25).

**Conclusion:** In our setting of random community surveillance, rapid antigen testing of nasopharyngeal swabs by either LIAISON SARS-CoV-2 Ag (DiaSorin) or Standard Q COVID-19 Ag (SD Biosensor) was less sensitive to detecting SARS-CoV-2 than the TaqPath COVID-19 RT-PCR.

## Introduction

Severe acute respiratory syndrome-related coronavirus 2 (SARS-CoV-2) caused a pandemic of respiratory illness, coronavirus disease 2019 (COVID-19), which exerted unprecedented pressure on healthcare systems around the world as well as on global economy (1, 2). Public health measures that include universal masking, early detection of infected individuals, isolation, and contact tracing have been the mainstay preventive strategy. Despite the immunization efforts, delays in vaccination role out, and emergence of novel variants leads to continued transmission and increase in COVID-19-related morbidity and mortality.

Polymerase chain reaction has been the gold-standard diagnostic method for SARS-CoV-2 infection (3). However, it is expensive, has a relative long turn-around-time, and require special laboratory set-up with fixed laboratory capacity. The need for larger test capacity and rapid case identification lead to the adoption of a point-of-care (POC) rapid antigen test in some screening programs (4). The test is cheap, rapid, easy to use, and does not require a laboratory setting (5). These factors allow the rapid antigen testing to overcome several logistical hurdles with mass SARS-CoV-2 testing. However, variation in test performance when used in different settings has been raised (3, 4, 6, 7).

Since the beginning of the pandemic and to better estimate disease activity and SARS-CoV-2 transmission, the Department of Public Health (Ministry of Health, Kuwait) conducted random community screening. Here, we assessed the performance of two antigen tests in detecting SARS-CoV-2 among individuals who were randomly selected in the community by the screening program.

## Methods

### Study Population and Selection

Individuals identified by the national COVID-19 random screening program conducted by the Public Health Department, Ministry of Health, were approached to participate in the study. The program includes testing units that approach household selected randomly using the Public Authority for the Civil Information (PACI) residential-units database. One individual per household is typically tested. The first consecutive ninety individuals were recruited into the study per day by a single testing unit over 10 days between May 24 and August 12, 2021. The recruitment days were determined by the working schedule of the testing units and the availability of the rapid antigen tests. The participating individuals were asked to fill a short questionnaire about the presence of respiratory symptoms, preexisting comorbidities, previous SARS-CoV-2 infection, and history of COVID-19 vaccination.

### Sample Collection and Transfer

Using polyester-tipped 3-dimentionally printed swabs, nasopharyngeal samples were collected by trained healthcare workers following a standard sample procurement procedure (8, 9). Two swabs were collected, one from each nostril, from each participating individual. One swab will be used for the point-of-care rapid on-site rapid antigen testing, while the second swab is used for the laboratory-based assays. Collected samples intended for laboratory-based tests were stored at a site and transported at 2–8°C immediately to the Jaber Innovation Laboratory at Jaber Alahmad Hospital and were processed within 12 h. If sample testing was expected to be delayed beyond 48 h, it was frozen at −70°C until processing. A single freeze-thaw cycle was allowed for PCR testing and the chemiluminescence-based assay according to the manufacturer's recommendations.

### SARS-CoV-2 Antigen Tests

Two SARS-CoV-2 antigen tests were used on each sample. Standard Q COVID-19 Ag test (SD Biosensor, Gyeonggi-do, Republic of Korea) is a POC chromatographic immunoassay that was performed by trained personnel immediately after sample collection. After obtaining the sample, the swab was placed in a supplied extraction buffer tube. It was then stirred five times and removed while squeezing the tube. Three drops of the solution were placed in the specimen well of the test device. After between 15 and 30 min, the result will be read and verified by two personnel. The card is read in a well-lit area, and the presence of any line at both the test and control marks was considered positive. If the control line was absent, the test was considered invalid and the sample was excluded from the analysis.

The second assay was the LIAISON SARS-CoV-2 Ag (DiaSorin, Saluggia, Italy), which is a laboratory-based chemiluminescence immunoassay (CLIA). One milliliter of the universal transport media containing the sample swab was mixed with an inactivation buffer following the manufacturer's recommendation. Assay procedure was performed using LIAISON® XL, a fully automated chemiluminescence analyzer. Both antigen assays that were used in this study detect the viral nuceleocapsid protein. Sample storage and processing were done according to the manufacturer's protocols.

### Nucleic Acid Extraction and Amplification Test

Sample extraction and nucleic acid purification were performed using the MagMAX™ kits and KingFisher Flex system (Thermofisher, MA, USA) and the virus DNA/RNA Extraction Kit using the Purifier HT extractor (Genfine, Beijing, China). SARS-CoV-2 RT-PCR was performed on extracted specimens, targeting the ORF, N, and S genes using the TaqPath™ COVID-19 RT PCR kit (Applied Biosystems, Bleiswijk, the Netherlands) using the Quant Studio 5 PCR system (Thermofisher, MA, USA). Result interpretations followed the manufacturer's recommendation. An inconclusive result was called when a single gene target was positive. RNA extraction and PCR were repeated in all inconclusive samples to confirm the result.

### Statistical Analysis

Demographic and clinical data were analyzed using descriptive statistics. Diagnostic performance of antigen assays with the corresponding 95% CI was calculated with PCR as the reference standard test. Result agreement was assessed using Cohen's kappa coefficient. Sensitivity analyses were done by restricting a positive result to individuals with a low-cycle threshold (Ct) value by PCR and to symptomatic individuals only. Low Ct value was considered in a sample with a value <25 in any of the targeted genes. All analyses were performed using STATA/IC 16 (STATA Corp, Texas).

### Ethics

The study protocol was approved by the Standing Committee for Coordination of Health and Medical Research (Ethics Review Committee) at the Ministry of Health (MOH) of Kuwait (reference No. 1566/2020).

## Results

**Figure 1:**
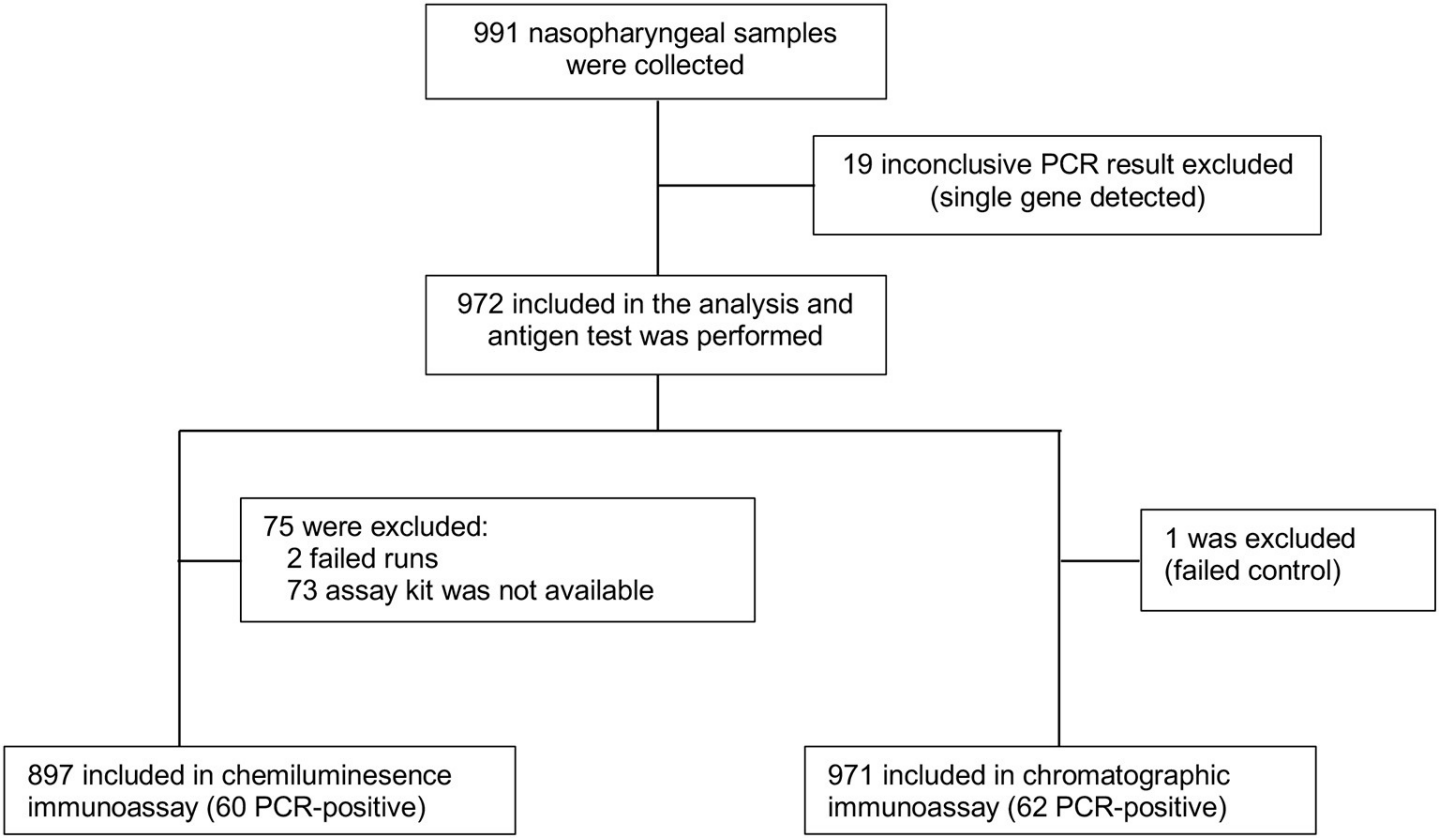
A flow diagram of the study population.

During the study period, 991 individuals agreed to participate in the study. A single PCR target was detected in 19 samples, was confirmed with repeat testing, and was excluded from the analysis (Figure 1). Of those sample, 15 had amplification of the N gene only, three were ORF gene positive, and one sample was S gene positive. In addition, one sample had a failed control and was excluded from Standard QAg test analysis. Also, 75 samples were excluded from the LIAISON SARS-CoV-2 Ag assay performance analysis (two samples failed controls, 73 were not done due to shortage of testing kits).

The median age of the participants was 40.2 years (IQR 32.3–47.8), and 932 (94%) were males. The high proportions of males can be attributed to the inclusion of low-skilled worker residential areas during the study period. Sixty-three subjects had preexisting medical conditions; of which, diabetes (53.9%) and hypertension (38.1%) were the most common conditions (Table 1). Quarter of individuals were fully or partially vaccinated against SARS-CoV-2, and 28 had previous infection. Minority of the participants (*n* = 30, 3%) had respiratory symptoms at the time of sample collection.

**Table 1:** Demographic and clinical data of the study population.

**Variable**	***n* (%) (*n* = 991)**
**Age** (median, IQR) in years	40.2 (32.3–47.8)
**Male**	932 (94.0%)
**Preexisting medical conditions**	63 (6.3)
Diabetes	34 (53.9)
Hypertension	24 (38.1)
Asthma	3 (4.7)
Missing	4 (6.3)
Other	5 (7.9)
**Previous SARS-CoV-2 infection**	28 (2.8)
**Vaccinated against COVID-19**	248 (25)
**Presence of respiratory symptoms at the time of testing**	30 (30)
Cough	17 (56.6)
Difficulty breathing	2 (6.6)
Fever	16 (53.3)
Loss of taste/smell	1 (3.3)
Muscle ache	4 (13.3)
Upper respiratory symptoms	2 (6.6)
**Positive SARS-CoV-2 PCR**	62 (6.2)
Asymptomatic infection	48 (77.4)

Sixty-two individuals (6.2%) had PCR-confirmed SARS-CoV-2 infection. Of those, 48 were asymptomatically infected. Median cycle threshold (Ct) was 22.6, 25.7, and 29.4 for S, ORF, and N genes, respectively. Patients with symptomatic COVID-19 had lower Ct values (19.9 for S gene, 19.1 for ORF gene, and 20.1 for N gene). Ct value of <25 in any of the gene targets was observed in 33 infected individuals.

All respiratory samples collected were tested using the chromatographic Standard Q assay. The control failed for one sample and was excluded from the analysis. Concordant results were observed in 94.4% of the samples and resulted in a fair agreement (Cohen's kappa 0.39). The assay was highly specific (98.8%, 95% CI, 97.8–99.4), but the sensitivity was low (30.6%, 95% CI, 19.6–43.7) (Table 2). On the other hand, LIAISON antigen detection had a moderate agreement (Cohen's kappa 0.58) with 96.1% of results being concordant. When compared to the Standard Q test, it was more sensitive (43.3%, 95% CI, 30.6–56.8) and specific (99.9%, 95% CI, 99.3–100). In addition, the LIAISON assay had higher positive predictive value (PPV) (96.3%; 95% CI, 81–99.9 vs. 63.3%; 95% CI, 43.9–80.1%).

**Table 2:** Characteristics of the **(A)** LIAISON SARS-CoV-2 Ag assay and **(B)** Standard Q antigen assays in the detection of SARS-CoV-2.

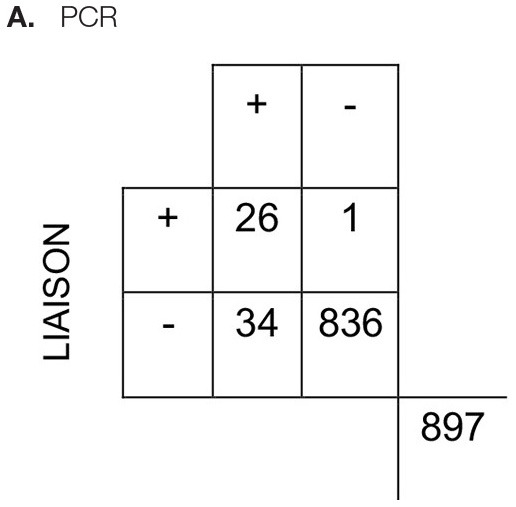				Estimate (%)	95% CI
			Sensitivity	43.3	30.6–56.8
			Specificity	99.9	99.3–100
			Positive predictive value	96.3	81–99.9
			Negative predictive value	96.1	94.6–97.3
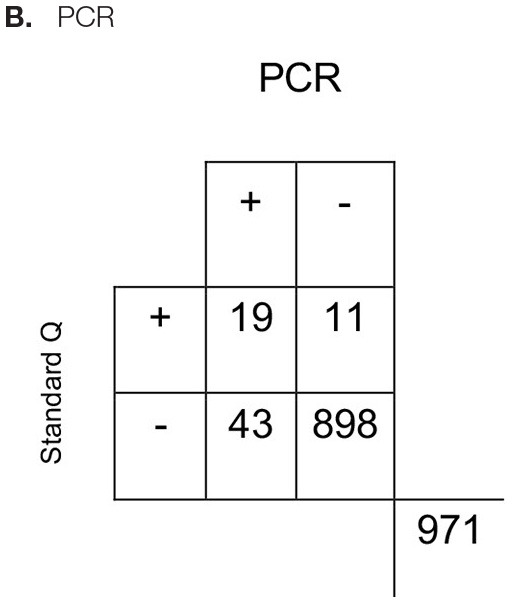				Estimate (%)	95% CI
			Sensitivity	30.6	19.6–43.7
			Specificity	98.8	97.8–99.4
			Positive predictive value	63.3	43.9–80.1
			Negative predictive value	95.4	93.9–96.7

Both assays performed better in symptomatic individuals and samples with low Ct values. The sensitivity of the LIAISON assay increased to 78.8% (95% CI, 61.1–91%) and 88.9% (95% CI, 51.8–99.7) when the analysis was limited to samples with low Ct value and symptomatic patients, respectively. Among the same subgroups, the PPV increased to 96.3% (95% CI, 81–99.9%) and 100% (95% CI, 63.1–100%). Similarly, the sensitivity of the Standard Qassay increased to 54.5% (95% CI, 36.4–71.9%) and 77.8% (95% CI, 40–97.2%) in a sample with low Ct value and symptomatic individuals. The PPV increased to 60% (95% CI, 40.6–77.3) and 87.5% (95% CI, 47.3–99.7%) (Appendix).

## Discussion

In community surveillance, an antigen detection test was less sensitive to detecting SARS-CoV-2. When compared to PCR, the chromatographic POC immunoassay (Standard Q) and CLIA (LIAISON) had a sensitivity of 30.1 and 45.6%, respectively. The performance of both assays improved with higher viral load as evident by Ct value and having symptomatic infection. Also, we found that using an antigen test for surveillance purposes may miss more than half of PCR-confirmed cases.

Antigen detection is highly dependent on the viral load in the specimen. We found that the sensitivity of both immunoassays is higher in symptomatic individuals and positive samples with low Ct value. In a University SARS-CoV-2 screening program, the positive agreement between Standard QAg test and PCR increased from 57.1 to 95.8% when the Ct value cutoff was decreased from 33 to 23 (3). These findings are in line with studies assessed by the analytical sensitivity of rapid antigen tests. The limit of detection of commercially available rapid antigen tests ranged between a Ct value equivalent of 18.4–30 (6, 10, 11). PCR can remain positive for a considerable period of time beyond the infectivity period (12). In a community screening program, the impact of missing individuals who are infected with low viral load is unknown. Also, preanalytical factors that may lead to lower viral load (e.g., sample collection technique, storage condition, delays in processing) may result in a false negative result.

In this study, we found that the automated CLIA-based LIAISON SARS-CoV-2 Ag assay had higher sensitivity with comparable specificity compared to the Standard QPOC chromatographic immunoassay. Also, the positive predictive value was higher in the CLIA (96.3 vs. 63.3%). Several factors can contribute to this discrepancy. One reason is that lateral flow assays, in general, have a higher lower limit of detection compared to other serological methods (13). Also, manufacturing technique and selection of assay design and material may greatly affect the test sensitivity (14). Furthermore, low viral load or poor analyte binding can result in a faint test line and may lead to poor result read-out. Lastly, the Food and Drug Administration listed cross-contamination and non-adherence to the manufacturer's instruction as the main contributor to false results (15). However, in this study, the Standard Qassay was performed immediately after interviewing each individual participant. In addition, all healthcare professionals received appropriate training in techniques to avoid cross contamination, importance of waiting the specified time before reading the result, and worked using the POC test in pairs.

This prospective study has few limitations. First, due to the design of the data collection form, the duration of symptoms was not consistently collected, resulting in significant missing data. However, the Ct value can be used as an indirect measure of the viral load and, hence, the duration of illness. Also, the samples were collected mostly during the beginning of a COVID-19 wave. This resulted in relatively low prevalence, which may greatly affect the predictive value of the tests. Yet, this finding may highlight the need for PCR confirmation in settings with low SARS-CoV-2 transmission. Also, this study adds to the growing evidence of the performance of different rapid SARS-CoV-2 antigen detections, especially in the community setting. In settings with low SARS-CoV-2 activity, the proportion of false negative results is high, and a negative test may warrant PCR confirmation.

## Data Availability Statement

The raw data supporting the conclusions of this article will be made available by the authors, without undue reservation.

## Ethics Statement

The study protocol was approved by the Standing Committee for Coordination of Health and Medical Research (Ethics Review Committee) at the Ministry of Health (MOH) of Kuwait (reference no. 1566/2020). The patients/participants provided their written informed consent to participate in this study.

## Author Contributions

MA and HB: conceptualization and methodology. MA, HB, FB, and HM: investigation, data curation, writing—original draft and review. HB: formal analysis and editing. SA-S: conceptualization, funding acquisition, supervision, writing, and review. All authors contributed to the article and approved the submitted version.

## Funding

This project was funded by the Kuwait Foundation for the Advancement of Science (KFAS) (Project code: PN20-15EI-01). Bader Sultan Medical Company and Innomedics Medical Company provided the SARS-CoV-2 antigen kits. All funding sources had no involvement in the study design or writing the manuscript.

## Conflict of Interest

The authors declare that the research was conducted in the absence of any commercial or financial relationships that could be construed as a potential conflict of interest.

## Publisher's Note

All claims expressed in this article are solely those of the authors and do not necessarily represent those of their affiliated organizations, or those of the publisher, the editors and the reviewers. Any product that may be evaluated in this article, or claim that may be made by its manufacturer, is not guaranteed or endorsed by the publisher.
