# Supplementary material for: The Performance of Two Rapid Antigen Tests During Population-Level Screening for SARS-CoV-2 Infection
Source: Front Med (Lausanne). 2021 Dec 23;8:797109. doi: 10.3389/fmed.2021.797109 (PMC8733308; doi:10.3389/fmed.2021.797109)
Supplement: Supplementary file 1 [file Table_1.docx]

**Supplementary material**

**Appendix table 1.** Characteristics of the (A.) LIAISON SARS-CoV-2 Ag assay and (B.) Standard Q antigen assays in the detection of SARS-CoV-2 considering low cycle threshold (Ct) value of less than 25 to be positive by polymerase chain reaction (PCR)

| **A.** |  | PCR | |  |  |  | Estimate (%) | 95% CI |
| --- | --- | --- | --- | --- | --- | --- | --- | --- |
|  |  | + | - |  |  | Sensitivity | 78.8 | 61.1 – 91.0 |
| LIAISON assay | + | 26 | 1 |  |  | Specificity | 99.9 | 99.4 – 100 |
|  | - | 7 | 847 |  |  | Positive predictive value | 96.3 | 81.0 – 99.9 |
|  |  |  |  | 897 |  | Negative predictive value | 99.2 | 98.3 – 99.7 |

| **B.** |  | PCR | |  |  |  | Estimate (%) | 95% CI |
| --- | --- | --- | --- | --- | --- | --- | --- | --- |
|  |  | + | - |  |  | Sensitivity | 54.5 | 36.4 – 71.9 |
| Standard Q | + | 18 | 12 |  |  | Specificity | 98.7 | 97.8 – 99.3 |
|  | - | 15 | 926 |  |  | Positive predictive value | 60.0 | 40.6 – 77.3 |
|  |  |  |  | 971 |  | Negative predictive value | 98.4 | 97.4 – 99.1 |

**Appendix table 2.** Characteristics of the (A.) LIAISON SARS-CoV-2 Ag assay and (B.) Standard Q antigen assays in the detection of SARS-CoV-2 among symptomatic patients only

| **A.** |  | PCR | |  |  |  | Estimate (%) | 95% CI |
| --- | --- | --- | --- | --- | --- | --- | --- | --- |
|  |  | + | - |  |  | Sensitivity | 88.9 | 51.8 – 99.7 |
| LIAISON assay | + | 8 | 0 |  |  | Specificity | 100 | 80.5 – 100 |
|  | - | 1 | 17 |  |  | Positive predictive value | 100 | 63.1 – 100 |
|  |  |  |  | 26 |  | Negative predictive value | 94.4 | 72.7 – 99.9 |

| **B.** |  | PCR | |  |  |  | Estimate (%) | 95% CI |
| --- | --- | --- | --- | --- | --- | --- | --- | --- |
|  |  | + | - |  |  | Sensitivity | 77.8 | 40 – 97.2 |
| Standard Q | + | 7 | 1 |  |  | Specificity | 94.7 | 74 – 99.9 |
|  | - | 2 | 18 |  |  | Positive predictive value | 87.5 | 47.3 – 99.7 |
|  |  |  |  | 28 |  | Negative predictive value | 90 | 68.3 – 98.8 |
